# Diethyl [(2-chloro­anilino)(1,3-diphenyl-1*H*-pyrazol-4-yl)meth­yl]phospho­nate

**DOI:** 10.1107/S1600536812051719

**Published:** 2013-01-04

**Authors:** G. Suresh, A. Nandakumar, V. Sabari, P. T. Perumal, S. Aravindhan

**Affiliations:** a Department of Physics, Presidency College (Autonomous), Chennai 600 005, India; b Organic Chemistry Laboratory, CLRI, Chennai, Tamilnadu, India

## Abstract

In the title compound, C_26_H_27_ClN_3_O_3_P, the mean plane of the central pyrazole ring forms a dihedral angle of 71.37 (14)° with the chloro­phenyl ring. In the crystal, mol­ecules are linked by pairs of N—H⋯O hydrogen bonds, forming inversion dimers with *R* ^2^_2_(10) ring motifs. The 3-phenyl ring is disordered with four C atoms occupying two sets of sites with an occupancy ratio of 0.748 (4):0.252 (4).

## Related literature

For information on pyrazole derivatives, see: Sullivan *et al.* (2006 ▶); Patel *et al.* (2010 ▶). For related structures, see: Saeed *et al.* (2009 ▶); Suresh *et al.* (2012 ▶). For hydrogen-bond motifs, see: Bernstein *et al.* (1995 ▶).
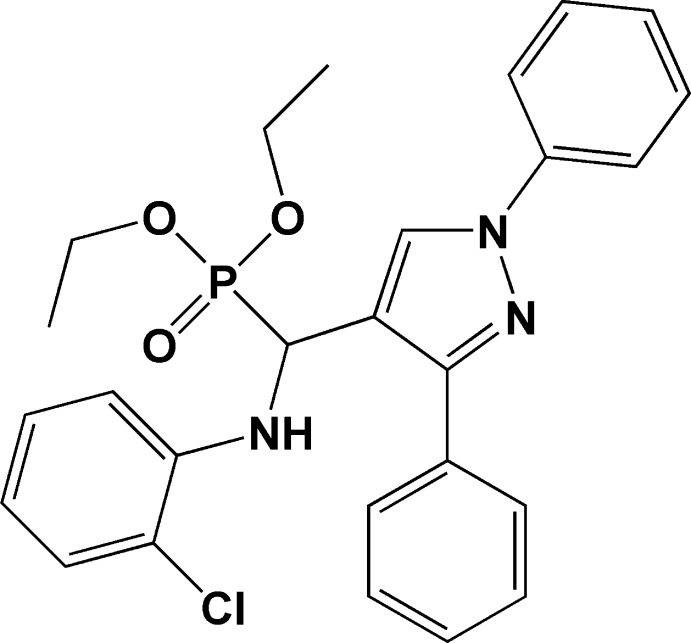


## Experimental

### 

#### Crystal data

C_26_H_27_ClN_3_O_3_P
*M* *_r_* = 495.93
Monoclinic, 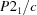

*a* = 11.2379 (3) Å
*b* = 23.7075 (6) Å
*c* = 9.4570 (2) Å
β = 90.809 (1)°
*V* = 2519.31 (11) Å^3^
*Z* = 4
Mo *K*α radiation
μ = 0.25 mm^−1^
*T* = 298 K
0.25 × 0.20 × 0.18 mm

#### Data collection

Bruker APEXII CCD area-detector diffractometer
Absorption correction: multi-scan (*SADABS*; Bruker, 2008 ▶) *T*_min_ = 0.941, *T*_max_ = 0.957
14995 measured reflections
4289 independent reflections
3322 reflections with *I* > 2σ(*I*)
*R*_int_ = 0.020

#### Refinement

*R*[*F* ^2^ > 2σ(*F* ^2^)] = 0.045
*wR*(*F* ^2^) = 0.135
*S* = 1.05
4289 reflections
346 parameters
99 restraints
H-atom parameters constrained
Δρ_max_ = 0.35 e Å^−3^
Δρ_min_ = −0.29 e Å^−3^

### 

Data collection: *APEX2* (Bruker, 2008 ▶); cell refinement: *SAINT* (Bruker, 2008 ▶); data reduction: *SAINT*; program(s) used to solve structure: *SHELXS97* (Sheldrick, 2008 ▶); program(s) used to refine structure: *SHELXL97* (Sheldrick, 2008 ▶); molecular graphics: *ORTEP-3* (Farrugia, 2012 ▶); software used to prepare material for publication: *SHELXL97* and *PLATON* (Spek, 2009 ▶).

## Supplementary Material

Crystal structure: contains datablock(s) I, global. DOI: 10.1107/S1600536812051719/su2545sup1.cif

Structure factors: contains datablock(s) I. DOI: 10.1107/S1600536812051719/su2545Isup2.hkl

Supplementary material file. DOI: 10.1107/S1600536812051719/su2545Isup3.cml

Additional supplementary materials:  crystallographic information; 3D view; checkCIF report

## Figures and Tables

**Table 1:** Hydrogen-bond geometry (Å, °)

*D*—H⋯*A*	*D*—H	H⋯*A*	*D*⋯*A*	*D*—H⋯*A*
N3—H3*A*⋯O1^i^	0.86	2.37	3.199 (3)	163

Symmetry code: (i) 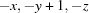.

**Fig. 1.**
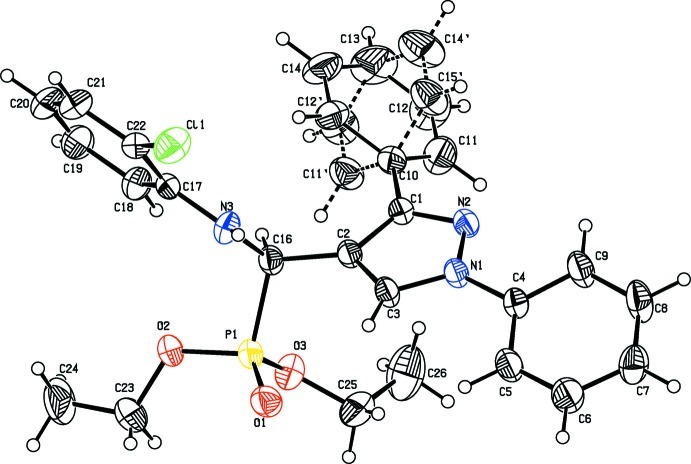
The molecular structure of the title molecule, with atom numbering. Displacement ellipsoids are drawn at the 30% probability level. Four C atoms in phenyl ring (C10-C16) are disordered over two positions (C11/C11', C12/C12', C14/C14' and C15/C15') with refined occupancies of 0.748 (4)/0.252 (4).

**Fig. 2.**
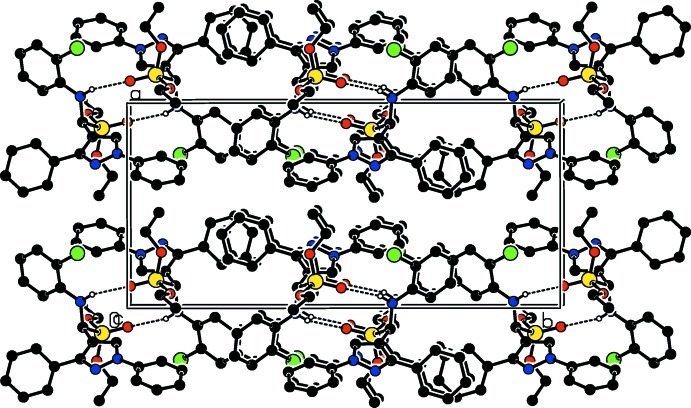
A view along the c axis of the crystal packing of the title compound, showing the formation of the inversion dimers (N-H···O hydrogen bonds are drawn as dashed lines).

## supplementary crystallographic information

### Comment

Pyrazoles exhibit a variety of pharmacological properties for e.g antibacterial and anti-inflammatory activities (Sullivan *et al.*, 2006; Patel *et al.*, 2010). In view of their importance, the title compound was synthesized and we report herein on its crystal structure.

The molecular structure of the title molecule is illustrated in Fig. 1. The bond lengths N2—C13 and N3—C14 are normal and comparable to the corresponding values observed in the related structure of 3-(3-Chloroanilino)-1-(3,5-dimethyl-1*H*-pyrazol-1-yl)propan-1-one (Saeed *et al.*, 2009). The pyrazole ring system is essentially planar, with a maximum deviation of -0.003 (2) Å for atom N2. The mean plane of the pyrazole ring and the chlorophenyl ring (C17-C22) are almost perpendicular to one another with a dihedral angle of 71.37 (14) °, whereas the two phenyl rings (C4-C9) and the major component of ring (C10-C15) are twisted out of the plane of the pyrazole ring, as can be seen from the dihedral angles of 15.84 (14)° and 39.2 (2)°, respectively.

The sum of the bond angles around atom N2 [359.75 (2)°] of the pyrazole ring is in accordance with sp^3^ hybridization. Atoms Cl1 and N3 deviate by -0.0278 (9) Å and 0.0206 (21) Å from the mean plane of the benzene (C17—C22) ring. The four carbon atoms in the phenyl ring (C10-C16) are disordered over two sets of sites [site occupancies = 0.748 (4) and 0.252 (4)]. The phosphinite group assumes an extended conformation as can be seen from the torsion angles P1—O2—C23—C24 of 179.2 (3)° and P1—O3—C25—C26 of 131.2 (2)°. They are close to those observed in a similar structure (Suresh *et al.*, 2012).

In the crystal, a pair of N—H···O hydrogen bonds link molecules to form inversion dimers, with an *R*^2^_2_(10) ring motif (Bernstein *et al.*, 1995), that stack along the c axis (Fig. 2 and Table 1).

### Experimental

A mixture of 1,3-diphenyl-1*H*-pyrazole-4-carbaldehyde (1 mmol), 2-chloroaniline (1 mmol), diethyl phosphite (1.5 mmol), and pottasium hydrogen sulfate (20 mol%) under neat condition was stirred at room temperature. After completion of the reaction as indicated by TLC, it was poured into water and extracted with ethyl acetate. The organic layer was dried over sodium sulfate and concentrated under vacuum. The crude product was chromatographed using an ethyl acetate/petroleum ether (30:70) mixture. Crystals suitable for X-ray diffraction were obtained by slow evaporation of a solution of the title compound in ethyl acetate at room temperature.

### Refinement

Four carbon atoms in phenyl ring (C10-C16) are disordered over two positions (C11/C11', C12/C12', C14/C14' and C15/C15') with refined occupancies of 0.748 (4)/0.252 (4). All C-bound H atoms were fixed geometrically and allowed to ride on their parent atom: C—H = 0.93–0.97 Å with *U*_iso_(H) = 1.5U_eq_(C) for methyl H atoms and = 1.2U_eq_(C) for other H atoms.

### Crystal data

**Table:**

C_26_H_27_ClN_3_O_3_P	*F*(000) = 1040
*M**_r_* = 495.93	*D*_x_ = 1.308 Mg m^−^^3^
Monoclinic, *P*2_1_/*c*	Mo *K*α radiation, λ = 0.71073 Å
Hall symbol: -P 2ybc	Cell parameters from 4289 reflections
*a* = 11.2379 (3) Å	θ = 1.8–25.0°
*b* = 23.7075 (6) Å	µ = 0.25 mm^−^^1^
*c* = 9.4570 (2) Å	*T* = 298 K
β = 90.809 (1)°	Monoclinic, colourless
*V* = 2519.31 (11) Å^3^	0.25 × 0.20 × 0.18 mm
*Z* = 4	

### Data collection

**Table:**

Bruker APEXII CCD area-detector diffractometer	4289 independent reflections
Radiation source: fine-focus sealed tube	3322 reflections with *I* > 2σ(*I*)
Graphite monochromator	*R*_int_ = 0.020
ω and φ scans	θ_max_ = 25.0°, θ_min_ = 1.8°
Absorption correction: multi-scan (*SADABS*; Bruker, 2008)	*h* = −12→13
*T*_min_ = 0.941, *T*_max_ = 0.957	*k* = −27→27
14995 measured reflections	*l* = −11→8

### Refinement

**Table:**

Refinement on *F*^2^	Primary atom site location: structure-invariant direct methods
Least-squares matrix: full	Secondary atom site location: difference Fourier map
*R*[*F*^2^ > 2σ(*F*^2^)] = 0.045	Hydrogen site location: inferred from neighbouring sites
*wR*(*F*^2^) = 0.135	H-atom parameters constrained
*S* = 1.05	*w* = 1/[σ^2^(*F*_o_^2^) + (0.0609*P*)^2^ + 1.4582*P*] where *P* = (*F*_o_^2^ + 2*F*_c_^2^)/3
4289 reflections	(Δ/σ)_max_ = 0.001
346 parameters	Δρ_max_ = 0.35 e Å^−^^3^
99 restraints	Δρ_min_ = −0.29 e Å^−^^3^

### Special details

**Table:**

Geometry. All e.s.d.'s (except the e.s.d. in the dihedral angle between two l.s. planes) are estimated using the full covariance matrix. The cell e.s.d.'s are taken into account individually in the estimation of e.s.d.'s in distances, angles and torsion angles; correlations between e.s.d.'s in cell parameters are only used when they are defined by crystal symmetry. An approximate (isotropic) treatment of cell e.s.d.'s is used for estimating e.s.d.'s involving l.s. planes.
Refinement. Refinement of *F*^2^ against ALL reflections. The weighted *R*-factor *wR* and goodness of fit *S* are based on *F*^2^, conventional *R*-factors *R* are based on *F*, with *F* set to zero for negative *F*^2^. The threshold expression of *F*^2^ > σ(*F*^2^) is used only for calculating *R*-factors(gt) *etc*. and is not relevant to the choice of reflections for refinement. *R*-factors based on *F*^2^ are statistically about twice as large as those based on *F*, and *R*- factors based on ALL data will be even larger.

### Fractional atomic coordinates and isotropic or equivalent isotropic displacement parameters (Å^2^)

**Table:**

	*x*	*y*	*z*	*U*_iso_*/*U*_eq_	Occ. (<1)
C1	0.2516 (2)	0.38895 (10)	0.2029 (2)	0.0451 (5)	
C2	0.1701 (2)	0.41684 (10)	0.1112 (2)	0.0446 (5)	
C3	0.1721 (2)	0.47164 (11)	0.1545 (2)	0.0493 (6)	
H3	0.1278	0.5011	0.1155	0.059*	
C4	0.2883 (2)	0.52521 (10)	0.3362 (2)	0.0472 (6)	
C5	0.2621 (3)	0.57751 (12)	0.2825 (3)	0.0707 (8)	
H5	0.2154	0.5808	0.2010	0.085*	
C6	0.3046 (3)	0.62490 (13)	0.3490 (4)	0.0801 (10)	
H6	0.2858	0.6603	0.3126	0.096*	
C7	0.3747 (3)	0.62076 (13)	0.4687 (3)	0.0713 (8)	
H7	0.4067	0.6529	0.5110	0.086*	
C8	0.3967 (3)	0.56867 (14)	0.5246 (3)	0.0785 (10)	
H8	0.4420	0.5656	0.6073	0.094*	
C9	0.3528 (3)	0.52038 (13)	0.4605 (3)	0.0686 (8)	
H9	0.3666	0.4852	0.5008	0.082*	
C10	0.2882 (2)	0.32923 (11)	0.2039 (3)	0.0540 (6)	
C13	0.3581 (4)	0.21686 (16)	0.2042 (5)	0.1082 (13)	
H13	0.3818	0.1793	0.2048	0.130*	
C11	0.4035 (4)	0.31377 (19)	0.2283 (6)	0.0841 (14)	0.748 (4)
H11	0.4605	0.3414	0.2457	0.101*	0.748 (4)
C12	0.4372 (5)	0.2577 (2)	0.2276 (8)	0.1102 (19)	0.748 (4)
H12	0.5166	0.2483	0.2438	0.132*	0.748 (4)
C14	0.2368 (5)	0.23124 (18)	0.1783 (6)	0.0879 (14)	0.748 (4)
H14	0.1809	0.2031	0.1608	0.106*	0.748 (4)
C15	0.2025 (4)	0.28700 (16)	0.1793 (4)	0.0658 (11)	0.748 (4)
H15	0.1232	0.2967	0.1638	0.079*	0.748 (4)
C11'	0.3088 (8)	0.2986 (5)	0.0798 (12)	0.070 (3)	0.252 (4)
H11'	0.2980	0.3164	−0.0071	0.084*	0.252 (4)
C12'	0.3449 (8)	0.2426 (5)	0.0829 (16)	0.087 (4)	0.252 (4)
H12'	0.3596	0.2235	−0.0010	0.104*	0.252 (4)
C14'	0.3428 (10)	0.2451 (6)	0.3322 (18)	0.099 (4)	0.252 (4)
H14'	0.3563	0.2267	0.4178	0.119*	0.252 (4)
C15'	0.3071 (9)	0.3009 (5)	0.3293 (13)	0.078 (3)	0.252 (4)
H15'	0.2957	0.3198	0.4142	0.094*	0.252 (4)
C16	0.1013 (2)	0.39307 (10)	−0.0133 (2)	0.0470 (6)	
H16	0.1320	0.3551	−0.0314	0.056*	
C17	−0.0919 (2)	0.34143 (10)	−0.0260 (2)	0.0494 (6)	
C18	−0.0505 (3)	0.30056 (12)	−0.1201 (3)	0.0654 (7)	
H18	0.0252	0.3043	−0.1575	0.078*	
C19	−0.1197 (3)	0.25502 (13)	−0.1583 (3)	0.0782 (9)	
H19	−0.0898	0.2282	−0.2202	0.094*	
C20	−0.2315 (4)	0.24868 (15)	−0.1066 (4)	0.0876 (11)	
H20	−0.2776	0.2178	−0.1336	0.105*	
C21	−0.2758 (3)	0.28790 (14)	−0.0146 (4)	0.0790 (9)	
H21	−0.3521	0.2839	0.0208	0.095*	
C22	−0.2059 (2)	0.33367 (11)	0.0254 (3)	0.0577 (7)	
C23	0.0637 (4)	0.42448 (19)	−0.4309 (3)	0.1112 (15)	
H23A	0.0251	0.4611	−0.4308	0.133*	
H23B	0.1444	0.4297	−0.4637	0.133*	
C24	0.0018 (4)	0.3882 (2)	−0.5261 (3)	0.1217 (17)	
H24A	0.0476	0.3545	−0.5404	0.183*	
H24B	−0.0102	0.4071	−0.6150	0.183*	
H24C	−0.0740	0.3784	−0.4874	0.183*	
C25	0.3665 (3)	0.46024 (16)	−0.1595 (4)	0.0920 (11)	
H25A	0.3975	0.4777	−0.2441	0.110*	
H25B	0.3381	0.4900	−0.0982	0.110*	
C26	0.4602 (3)	0.4301 (2)	−0.0887 (6)	0.1312 (18)	
H26A	0.4325	0.4167	0.0009	0.197*	
H26B	0.5269	0.4547	−0.0737	0.197*	
H26C	0.4838	0.3986	−0.1456	0.197*	
Cl1	−0.26240 (7)	0.38210 (4)	0.14418 (9)	0.0802 (3)	
N1	0.24974 (17)	0.47557 (8)	0.26426 (19)	0.0464 (5)	
N2	0.30036 (17)	0.42479 (9)	0.2952 (2)	0.0489 (5)	
N3	−0.02512 (18)	0.38798 (9)	0.0135 (2)	0.0533 (5)	
H3A	−0.0602	0.4153	0.0559	0.064*	
O1	0.10231 (18)	0.49551 (8)	−0.15609 (19)	0.0643 (5)	
O2	0.06769 (17)	0.40281 (8)	−0.28883 (17)	0.0617 (5)	
O3	0.26813 (16)	0.42354 (9)	−0.1981 (2)	0.0670 (5)	
P1	0.13347 (6)	0.43618 (3)	−0.16802 (6)	0.0497 (2)	

### Atomic displacement parameters (Å^2^)

**Table:**

	*U*^11^	*U*^22^	*U*^33^	*U*^12^	*U*^13^	*U*^23^
C1	0.0444 (13)	0.0487 (14)	0.0421 (12)	−0.0027 (11)	−0.0041 (10)	0.0027 (10)
C2	0.0463 (13)	0.0481 (14)	0.0393 (11)	−0.0002 (11)	−0.0060 (9)	−0.0011 (10)
C3	0.0538 (14)	0.0513 (15)	0.0422 (12)	0.0051 (12)	−0.0145 (10)	−0.0016 (11)
C4	0.0485 (13)	0.0519 (15)	0.0410 (12)	0.0028 (11)	−0.0057 (10)	−0.0064 (11)
C5	0.094 (2)	0.0553 (18)	0.0615 (16)	−0.0018 (16)	−0.0321 (15)	0.0019 (14)
C6	0.108 (3)	0.0520 (18)	0.080 (2)	−0.0001 (17)	−0.0261 (19)	−0.0025 (15)
C7	0.077 (2)	0.0625 (19)	0.0740 (19)	0.0005 (16)	−0.0107 (15)	−0.0258 (16)
C8	0.094 (2)	0.075 (2)	0.0650 (18)	0.0122 (18)	−0.0344 (16)	−0.0233 (16)
C9	0.089 (2)	0.0608 (18)	0.0551 (15)	0.0094 (16)	−0.0290 (14)	−0.0082 (14)
C10	0.0607 (16)	0.0463 (15)	0.0547 (14)	−0.0004 (12)	−0.0087 (12)	0.0062 (12)
C13	0.126 (3)	0.052 (2)	0.146 (4)	0.020 (2)	−0.003 (3)	0.012 (2)
C11	0.074 (3)	0.058 (3)	0.119 (4)	0.006 (2)	−0.031 (3)	0.006 (2)
C12	0.090 (4)	0.069 (3)	0.171 (5)	0.019 (3)	−0.033 (4)	0.010 (3)
C14	0.103 (4)	0.048 (2)	0.113 (4)	−0.020 (2)	0.004 (3)	0.008 (2)
C15	0.067 (2)	0.057 (2)	0.074 (2)	−0.0103 (18)	0.0015 (19)	0.0105 (19)
C11'	0.090 (7)	0.056 (6)	0.063 (6)	0.011 (6)	−0.009 (5)	0.004 (5)
C12'	0.115 (8)	0.057 (7)	0.088 (7)	0.024 (6)	−0.009 (7)	−0.010 (6)
C14'	0.134 (9)	0.063 (7)	0.100 (8)	0.044 (7)	−0.006 (8)	0.017 (7)
C15'	0.111 (8)	0.059 (6)	0.064 (6)	0.026 (6)	−0.013 (6)	−0.002 (5)
C16	0.0518 (14)	0.0464 (14)	0.0426 (12)	0.0003 (11)	−0.0083 (10)	−0.0047 (11)
C17	0.0626 (15)	0.0428 (14)	0.0424 (12)	−0.0087 (12)	−0.0154 (11)	0.0064 (10)
C18	0.0791 (19)	0.0544 (17)	0.0624 (16)	−0.0110 (15)	−0.0067 (14)	−0.0093 (14)
C19	0.112 (3)	0.0500 (18)	0.0719 (19)	−0.0144 (18)	−0.0137 (19)	−0.0088 (15)
C20	0.119 (3)	0.061 (2)	0.082 (2)	−0.042 (2)	−0.013 (2)	0.0001 (18)
C21	0.085 (2)	0.073 (2)	0.079 (2)	−0.0352 (18)	−0.0042 (17)	0.0125 (18)
C22	0.0669 (17)	0.0539 (16)	0.0519 (14)	−0.0160 (13)	−0.0095 (12)	0.0121 (12)
C23	0.162 (4)	0.125 (3)	0.0452 (16)	−0.055 (3)	−0.026 (2)	0.0146 (18)
C24	0.128 (3)	0.189 (5)	0.0480 (18)	−0.047 (3)	−0.0108 (19)	−0.017 (2)
C25	0.078 (2)	0.090 (3)	0.108 (3)	−0.030 (2)	−0.012 (2)	0.015 (2)
C26	0.060 (2)	0.158 (5)	0.175 (5)	−0.002 (3)	−0.018 (3)	−0.027 (4)
Cl1	0.0734 (5)	0.0785 (6)	0.0892 (5)	−0.0131 (4)	0.0136 (4)	−0.0062 (4)
N1	0.0513 (11)	0.0467 (12)	0.0410 (10)	0.0031 (9)	−0.0103 (8)	−0.0026 (9)
N2	0.0524 (12)	0.0497 (12)	0.0442 (10)	0.0010 (10)	−0.0106 (9)	0.0028 (9)
N3	0.0511 (12)	0.0482 (12)	0.0604 (12)	−0.0072 (10)	−0.0047 (9)	−0.0126 (10)
O1	0.0819 (13)	0.0511 (11)	0.0597 (11)	0.0016 (9)	−0.0099 (9)	0.0017 (9)
O2	0.0740 (12)	0.0688 (12)	0.0420 (9)	−0.0130 (10)	−0.0130 (8)	−0.0015 (8)
O3	0.0575 (11)	0.0711 (13)	0.0722 (12)	−0.0085 (9)	−0.0037 (9)	−0.0109 (10)
P1	0.0539 (4)	0.0516 (4)	0.0433 (3)	−0.0042 (3)	−0.0092 (3)	−0.0033 (3)

### Geometric parameters (Å, º)

**Table:**

C1—N2	1.330 (3)	C14'—C15'	1.382 (17)
C1—C2	1.416 (3)	C14'—H14'	0.9300
C1—C10	1.474 (3)	C15'—H15'	0.9300
C2—C3	1.362 (3)	C16—N3	1.452 (3)
C2—C16	1.509 (3)	C16—P1	1.825 (2)
C3—N1	1.350 (3)	C16—H16	0.9800
C3—H3	0.9300	C17—N3	1.383 (3)
C4—C5	1.370 (4)	C17—C22	1.389 (4)
C4—C9	1.377 (3)	C17—C18	1.400 (4)
C4—N1	1.424 (3)	C18—C19	1.376 (4)
C5—C6	1.370 (4)	C18—H18	0.9300
C5—H5	0.9300	C19—C20	1.363 (5)
C6—C7	1.374 (4)	C19—H19	0.9300
C6—H6	0.9300	C20—C21	1.372 (5)
C7—C8	1.364 (4)	C20—H20	0.9300
C7—H7	0.9300	C21—C22	1.389 (4)
C8—C9	1.383 (4)	C21—H21	0.9300
C8—H8	0.9300	C22—Cl1	1.733 (3)
C9—H9	0.9300	C23—C24	1.421 (5)
C10—C11	1.363 (5)	C23—O2	1.439 (3)
C10—C15'	1.377 (12)	C23—H23A	0.9700
C10—C11'	1.401 (12)	C23—H23B	0.9700
C10—C15	1.406 (4)	C24—H24A	0.9600
C13—C12'	1.306 (15)	C24—H24B	0.9600
C13—C12	1.331 (7)	C24—H24C	0.9600
C13—C14'	1.396 (17)	C25—C26	1.430 (5)
C13—C14	1.422 (6)	C25—O3	1.450 (4)
C13—H13	0.9300	C25—H25A	0.9700
C11—C12	1.382 (6)	C25—H25B	0.9700
C11—H11	0.9300	C26—H26A	0.9600
C12—H12	0.9300	C26—H26B	0.9600
C14—C15	1.377 (6)	C26—H26C	0.9600
C14—H14	0.9300	N1—N2	1.361 (3)
C15—H15	0.9300	N3—H3A	0.8600
C11'—C12'	1.388 (17)	O1—P1	1.454 (2)
C11'—H11'	0.9300	O2—P1	1.5664 (17)
C12'—H12'	0.9300	O3—P1	1.573 (2)
			
N2—C1—C2	111.1 (2)	C13—C14'—H14'	120.6
N2—C1—C10	119.8 (2)	C10—C15'—C14'	121.7 (12)
C2—C1—C10	129.0 (2)	C10—C15'—H15'	119.1
C3—C2—C1	104.7 (2)	C14'—C15'—H15'	119.1
C3—C2—C16	126.6 (2)	N3—C16—C2	112.74 (19)
C1—C2—C16	128.6 (2)	N3—C16—P1	113.08 (16)
N1—C3—C2	107.7 (2)	C2—C16—P1	108.14 (16)
N1—C3—H3	126.1	N3—C16—H16	107.5
C2—C3—H3	126.1	C2—C16—H16	107.5
C5—C4—C9	119.9 (2)	P1—C16—H16	107.5
C5—C4—N1	120.6 (2)	N3—C17—C22	120.7 (2)
C9—C4—N1	119.5 (2)	N3—C17—C18	122.7 (2)
C4—C5—C6	120.0 (3)	C22—C17—C18	116.5 (2)
C4—C5—H5	120.0	C19—C18—C17	121.3 (3)
C6—C5—H5	120.0	C19—C18—H18	119.4
C5—C6—C7	120.8 (3)	C17—C18—H18	119.4
C5—C6—H6	119.6	C20—C19—C18	120.8 (3)
C7—C6—H6	119.6	C20—C19—H19	119.6
C8—C7—C6	118.8 (3)	C18—C19—H19	119.6
C8—C7—H7	120.6	C19—C20—C21	119.8 (3)
C6—C7—H7	120.6	C19—C20—H20	120.1
C7—C8—C9	121.2 (3)	C21—C20—H20	120.1
C7—C8—H8	119.4	C20—C21—C22	119.6 (3)
C9—C8—H8	119.4	C20—C21—H21	120.2
C4—C9—C8	119.0 (3)	C22—C21—H21	120.2
C4—C9—H9	120.5	C21—C22—C17	122.0 (3)
C8—C9—H9	120.5	C21—C22—Cl1	118.9 (2)
C11—C10—C15'	65.7 (5)	C17—C22—Cl1	119.2 (2)
C11—C10—C11'	80.5 (4)	C24—C23—O2	112.6 (3)
C15'—C10—C11'	116.3 (7)	C24—C23—H23A	109.1
C11—C10—C15	118.9 (3)	O2—C23—H23A	109.1
C15'—C10—C15	83.8 (5)	C24—C23—H23B	109.1
C11'—C10—C15	67.3 (4)	O2—C23—H23B	109.1
C11—C10—C1	121.6 (3)	H23A—C23—H23B	107.8
C15'—C10—C1	120.9 (6)	C23—C24—H24A	109.5
C11'—C10—C1	122.8 (5)	C23—C24—H24B	109.5
C15—C10—C1	119.5 (3)	H24A—C24—H24B	109.5
C12'—C13—C12	82.7 (5)	C23—C24—H24C	109.5
C12'—C13—C14'	121.6 (8)	H24A—C24—H24C	109.5
C12—C13—C14'	66.3 (6)	H24B—C24—H24C	109.5
C12'—C13—C14	68.9 (5)	C26—C25—O3	111.8 (3)
C12—C13—C14	119.4 (4)	C26—C25—H25A	109.3
C14'—C13—C14	84.6 (5)	O3—C25—H25A	109.3
C12'—C13—H13	118.8	C26—C25—H25B	109.3
C12—C13—H13	120.3	O3—C25—H25B	109.3
C14'—C13—H13	119.5	H25A—C25—H25B	107.9
C14—C13—H13	120.3	C25—C26—H26A	109.5
C10—C11—C12	121.2 (4)	C25—C26—H26B	109.5
C10—C11—H11	119.4	H26A—C26—H26B	109.5
C12—C11—H11	119.4	C25—C26—H26C	109.5
C13—C12—C11	121.2 (5)	H26A—C26—H26C	109.5
C13—C12—H12	119.4	H26B—C26—H26C	109.5
C11—C12—H12	119.4	C3—N1—N2	111.57 (19)
C15—C14—C13	119.8 (4)	C3—N1—C4	127.9 (2)
C15—C14—H14	120.1	N2—N1—C4	120.33 (18)
C13—C14—H14	120.1	C1—N2—N1	104.91 (18)
C14—C15—C10	119.5 (4)	C17—N3—C16	123.2 (2)
C14—C15—H15	120.2	C17—N3—H3A	118.4
C10—C15—H15	120.2	C16—N3—H3A	118.4
C12'—C11'—C10	122.0 (11)	C23—O2—P1	120.6 (2)
C12'—C11'—H11'	119.0	C25—O3—P1	124.9 (2)
C10—C11'—H11'	119.0	O1—P1—O2	115.68 (11)
C13—C12'—C11'	119.7 (12)	O1—P1—O3	115.58 (12)
C13—C12'—H12'	120.2	O2—P1—O3	102.56 (11)
C11'—C12'—H12'	120.2	O1—P1—C16	115.40 (11)
C15'—C14'—C13	118.7 (12)	O2—P1—C16	101.83 (10)
C15'—C14'—H14'	120.6	O3—P1—C16	103.89 (11)
			
N2—C1—C2—C3	−0.4 (3)	C11—C10—C15'—C14'	−65.7 (6)
C10—C1—C2—C3	−178.4 (2)	C11'—C10—C15'—C14'	−0.9 (6)
N2—C1—C2—C16	176.0 (2)	C15—C10—C15'—C14'	59.9 (6)
C10—C1—C2—C16	−2.0 (4)	C1—C10—C15'—C14'	−179.3 (5)
C1—C2—C3—N1	0.1 (3)	C13—C14'—C15'—C10	−0.9 (9)
C16—C2—C3—N1	−176.4 (2)	C3—C2—C16—N3	−74.9 (3)
C9—C4—C5—C6	3.0 (5)	C1—C2—C16—N3	109.4 (3)
N1—C4—C5—C6	−176.8 (3)	C3—C2—C16—P1	50.8 (3)
C4—C5—C6—C7	0.7 (5)	C1—C2—C16—P1	−124.8 (2)
C5—C6—C7—C8	−3.2 (5)	N3—C17—C18—C19	−179.3 (3)
C6—C7—C8—C9	2.0 (5)	C22—C17—C18—C19	−0.4 (4)
C5—C4—C9—C8	−4.1 (5)	C17—C18—C19—C20	0.7 (5)
N1—C4—C9—C8	175.7 (3)	C18—C19—C20—C21	−0.4 (5)
C7—C8—C9—C4	1.6 (5)	C19—C20—C21—C22	−0.2 (5)
N2—C1—C10—C11	−38.1 (4)	C20—C21—C22—C17	0.5 (5)
C2—C1—C10—C11	139.7 (4)	C20—C21—C22—Cl1	−178.9 (3)
N2—C1—C10—C15'	40.6 (5)	N3—C17—C22—C21	178.7 (2)
C2—C1—C10—C15'	−141.6 (5)	C18—C17—C22—C21	−0.2 (4)
N2—C1—C10—C11'	−137.6 (4)	N3—C17—C22—Cl1	−1.9 (3)
C2—C1—C10—C11'	40.2 (5)	C18—C17—C22—Cl1	179.18 (19)
N2—C1—C10—C15	141.8 (3)	C2—C3—N1—N2	0.3 (3)
C2—C1—C10—C15	−40.4 (4)	C2—C3—N1—C4	174.4 (2)
C15'—C10—C11—C12	68.1 (8)	C5—C4—N1—C3	−10.9 (4)
C11'—C10—C11—C12	−56.6 (7)	C9—C4—N1—C3	169.3 (3)
C15—C10—C11—C12	0.7 (7)	C5—C4—N1—N2	162.8 (3)
C1—C10—C11—C12	−179.4 (5)	C9—C4—N1—N2	−17.0 (4)
C12'—C13—C12—C11	61.3 (8)	C2—C1—N2—N1	0.6 (3)
C14'—C13—C12—C11	−68.0 (8)	C10—C1—N2—N1	178.8 (2)
C14—C13—C12—C11	0.5 (10)	C3—N1—N2—C1	−0.6 (3)
C10—C11—C12—C13	−0.5 (10)	C4—N1—N2—C1	−175.2 (2)
C12'—C13—C14—C15	−68.8 (8)	C22—C17—N3—C16	167.5 (2)
C12—C13—C14—C15	−0.7 (8)	C18—C17—N3—C16	−13.7 (4)
C14'—C13—C14—C15	58.1 (7)	C2—C16—N3—C17	−137.3 (2)
C13—C14—C15—C10	0.9 (7)	P1—C16—N3—C17	99.7 (2)
C11—C10—C15—C14	−0.9 (6)	C24—C23—O2—P1	179.2 (3)
C15'—C10—C15—C14	−58.7 (6)	C26—C25—O3—P1	131.2 (3)
C11'—C10—C15—C14	63.2 (6)	C23—O2—P1—O1	50.1 (3)
C1—C10—C15—C14	179.2 (3)	C23—O2—P1—O3	−76.7 (3)
C11—C10—C11'—C12'	57.5 (3)	C23—O2—P1—C16	176.0 (3)
C15'—C10—C11'—C12'	0.8 (3)	C25—O3—P1—O1	26.1 (3)
C15—C10—C11'—C12'	−69.5 (4)	C25—O3—P1—O2	152.9 (2)
C1—C10—C11'—C12'	179.1 (3)	C25—O3—P1—C16	−101.3 (2)
C12—C13—C12'—C11'	−59.7 (4)	N3—C16—P1—O1	65.96 (19)
C14'—C13—C12'—C11'	−3.4 (6)	C2—C16—P1—O1	−59.6 (2)
C14—C13—C12'—C11'	65.7 (4)	N3—C16—P1—O2	−60.18 (19)
C10—C11'—C12'—C13	1.4 (3)	C2—C16—P1—O2	174.24 (16)
C12'—C13—C14'—C15'	3.2 (9)	N3—C16—P1—O3	−166.47 (16)
C12—C13—C14'—C15'	67.5 (6)	C2—C16—P1—O3	67.95 (18)
C14—C13—C14'—C15'	−57.9 (6)		

### Hydrogen-bond geometry (Å, º)

**Table:**

*D*—H···*A*	*D*—H	H···*A*	*D*···*A*	*D*—H···*A*
N3—H3*A*···O1^i^	0.86	2.37	3.199 (3)	163

Symmetry code: (i) −*x*, −*y*+1, −*z*.
